# A review on toxic effects of pesticides in Zebrafish, *Danio rerio* and common carp, *Cyprinus carpio*, emphasising Atrazine herbicide

**DOI:** 10.1016/j.toxrep.2024.101694

**Published:** 2024-07-14

**Authors:** Zahra Khoshnood

**Affiliations:** Department of Biology, Dezful Branch, Islamic Azad University, Dezful, Iran

**Keywords:** Pesticides, Fish, Toxicity mechanisms, Herbicide, Exposure

## Abstract

The widespread use of pesticides has emerged as a pressing environmental concern nowadays. These chemical compounds pose a significant threat to aquatic organisms due to their toxic effects. Zebrafish and common carp are two common species used in pesticide toxicity studies. Atrazine, a widely used herbicide, is one of the most prevalent globally, detectable in nearly all surface waters. This article examines existing literature to provide a comprehensive review of the toxic effects of Atrazine on Zebrafish and common carp. The findings reveal that exposure to atrazine triggers a range of biochemical, physiological, behavioral, and genetic alterations in these fish species, even at concentrations deemed environmentally relevant. These changes could have severe consequences, including increased mortality rates, reproductive failures, and potentially leading to fish populations decline. It is, therefore, imperative to prioritize stringent regulatory measures to curb the usage of this herbicide and safeguard fish species as unintended victims of aquatic ecosystems.

## Highlights

• Atrazine has toxic effects on common carp and zebrafish tissues, enzymes and genes.
• Atrazine is toxic to non-target aquatic organisms even at very low concentrations.
• DNA alterations affected by atrazine may inherit to the next generation of fish.

## Graphical Abstract

**Figure:**
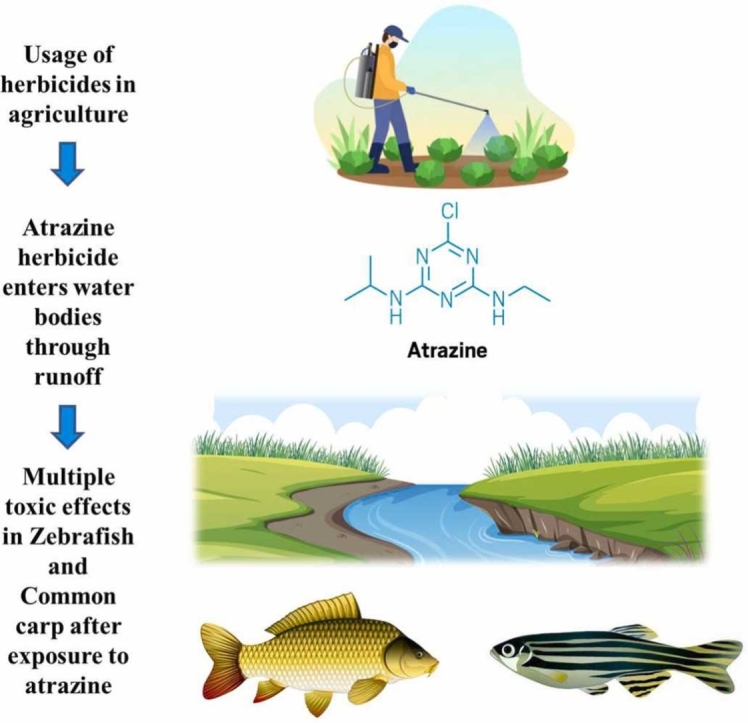

## 1 Introduction

Pesticides are chemical substances designed to control pests that damage crops and transmit diseases. Their usage has become globally widespread, facilitating increased agricultural productivity [15]. However, these chemicals have been associated with a various ecological and human health concerns, necessitating a deeper understanding of their impacts. The issue of pesticide residue hazards is increasingly alarming. Residual pesticides have the potential to seep into surface water, creating a significant risk to aquatic life [74].

The link between pesticides and declines in biodiversity includes the loss of beneficial insects, birds, and aquatic species [63]. Pesticide exposure can disrupt ecosystem functioning, leading to imbalances in predator-prey relationships and reduced pollination services [25]. Pesticides can also persist in the environment, accumulating in soil and water systems, thus posing long-term risks to terrestrial and aquatic organisms [48].

Runoff from agricultural fields can carry pesticide residues into nearby water bodies, leading to water contamination. In recent years, numerous researches have demonstrated the impact of pesticides on the aquatic environment [67], [21], [23], [73]. Pesticides such as organophosphates and neonicotinoids have been detected in streams, rivers, and groundwater, posing risks to aquatic organisms and affecting the freshwater ecosystems integrity [71].

When introduced into the environment, organophosphates, pyrethroids, and other chemicals can pose a significant threat to fish living in aquatic ecosystems [49]. Exposure to certain substances in aquatic environments can result in severe consequences for fish, including immediate mortality or disruptions to their behavior, feeding habits, swimming abilities, and reproductive capabilities.

Certain pesticides, particularly those with persistent characteristics, can accumulate in the fish tissues over time. This bioaccumulation can occur through direct exposure or by consuming contaminated prey. Pesticide residues can then reach levels with chronic health effects on fish and possibly transfer up the food chain [96], [95], [62].

Pesticide exposure can disrupt the reproductive processes of fish. Some pesticides act as endocrine disruptors, interfering with hormone signaling, which may lead to abnormalities in reproductive organs, reduced fertility, and altered mating behaviors [39]. Fish exposed to pesticides may exhibit slower growth rates and impaired development. Pesticides can interfere with metabolic processes, disrupt the balance of essential nutrients, and affect gene expression, thereby affecting the overall growth and development of fish populations [41], [54].

Pesticides can have neurological effects on fish, disrupting their sensory systems, cognition, and behavior. Fish exposed to pesticides may exhibit abnormal swimming patterns, reduced predator avoidance, and impaired foraging abilities, ultimately impacting their survival in natural habitats [8], [41], [72]. These chemicals contaminate aquatic habitats and can indirectly impact fish populations by altering the availability and quality of critical habitats [14]; for example, herbicides can eliminate vegetation cover that provides shelter and food resources for fish, leading to a decline in population abundance and diversity [28].

The effects of pesticide exposure on fish can be quite complex and involve a variety of genes. Some of the genes that have been found to be affected by pesticide exposure in fish include those involved in detoxification processes, such as cytochrome P450 enzymes (CYP), glutathione *S*-transferases, and UDP-glucuronosyltransferases. Additionally, pesticide exposure in fish may also impact genes related to oxidative stress response, immune function, and reproductive processes. The specific genes affected can vary depending on the type of pesticide, the dosage, and the fish species being studied [94], [79].

**Fig. 1:**
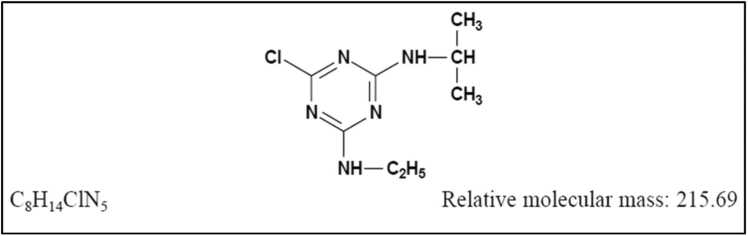
Atrazine chemical structure.

Atrazine is a synthetic herbicide that belongs to the class of triazine compounds. Chemically, it is known as 2-chloro-4-(ethylamino)-6-(isopropylamino)-S-triazine (Fig. 1). Atrazine is commercially available in various forms: such as dry flowable, flowable liquid, flowable suspension concentrate, liquid, water-dispersible granular, and wettable powder formulations. The technical-grade atrazine typically has a purity level between 92 % and 97 %. Atrazine commonly contains impurities such as dichloro triazines, tris(alkylamino)triazines, and hydroxy triazines [50]. The concentration of active ingredient in most atrazine products registered with the United States Environmental Protection Agency is 43, 80 or 90 %; the concentration in other atrazine products ranges from 0.58 % to 53.5 % [50]. Atrazine can be combined with various other herbicides, such as alachlor, acetochlor, ametryn, amitrole, benoxacor, bentazone, bromoxynil, cyanazine, 2,4-D, dicamba potassium salt, dichlobenil, diuron, glyphosate, imazapyr, imazethapyr, metolachlor, pendimethalin, pyridate, and simazine. It is commonly used in blends with these herbicides for spraying and mixed into fertilizer [50].

This powerful herbicide is widely used in agricultural practices to control broadleaf and grassy weeds, primarily in corn crops. Atrazine inhibits photosynthesis in susceptible plants, disrupting the electron transport process in chloroplasts. This ultimately leads to the death of target weeds (like annual bluegrass, white clover, chickweed, bedstraw and lawn burweed), allowing the desired crops to flourish. It is essential to point out that atrazine's effectiveness also comes with certain considerations. Due to its persistence in soil and moderate solubility in water, concerns have been about its potential impact on ecosystems, particularly in aquatic environments. Atrazine can be detected in nearly every surface water system in the world and has a half-life ranging from 2 to 800 days, depending on pH and other environmental factors. Concentrations of atrazine in the aquatic environment can exceed 20 mg l^−1^ [68], and a recent study reported the atrazine concentration of 0.17 μg L^−1^ in lakes of the United States [4]. Studies have suggested that atrazine runoff from fields can potentially contaminate water sources, affecting aquatic organisms and their habitats [38], [41], [16].

Zebrafish, *Danio rerio*, is a small tropical freshwater fish of the Cyprinidae family. They are native to the Southeast Asia and are often found in streams, ponds, and rice paddies. Zebrafish have become incredibly popular in scientific research due to their unique characteristics and genetic similarities to humans. They are transparent during their early stages of development, allowing scientists to observe their internal organs and processes easily. This transparency, combined with their relatively quick and simple reproduction, makes them an excellent model organism for studying various aspects of biology, genetics, and human diseases. They are also remarkably resilient and adaptable, tolerating a wide range of water conditions and temperatures [3], [11].

Common carp, *Cyprinus carpio* of the Cyprinidae family is also a species of freshwater fish native to Europe and Asia. They have been widely introduced to various parts of the world and are now found in many countries. In terms of their behavior, common carp are known to be highly adaptive and resilient. They can tolerate a wide range of water conditions and often habit in slow-moving rivers, ponds, and lakes. Due to low requirements, high compatibility, and high dispersion, common carp is a widely used fish as an experimental species in evaluating the effects of environmental contaminants, besides its commercial and ecological importance [32].

These species (*D. rerio* and *C. carpio*) are common fish investigated in various studies to determine the toxicity effects of pesticides as laboratory model organisms. These species are known in different countries and readily available to researchers, and they are considered valuable from the economic and ecological point of view. For this reason, the focus of this article is on the results of studies conducted on these two species.

As it mentioned before, the present manuscript aimed to review the toxic effects of pesticides, emphasizing atrazine, in two common fish species (*D. rerio* and *C. carpio*) as non-target aquatic organisms using literature reviews.

## 2 Toxicity on enzymes

### 2.1 Cytochrome P450

Pesticides can be metabolized by CYP enzymes in fish, leading to the formation of more toxic or less toxic metabolites. Changes in the activity of CYP enzymes can affect the rate at which pesticides are metabolized, potentially altering their toxicity [45].

CYP enzymes are also involved in metabolizing endogenous compounds, such as hormones and fatty acids. Disruption of CYP enzyme activity by pesticides alter the metabolism of these endogenous compounds, impacting various physiological processes in fish [94].

Some pesticides can induce oxidative stress in fish by generating reactive oxygen species. CYP enzymes are involved in both the generation and detoxification of reactive oxygen species, and alterations in CYP enzyme activity can impact the fish's ability to cope with oxidative stress induced by pesticides [53].

Changes in the activity of CYP enzymes can also influence the metabolism and toxicity of other chemicals that the fish may be exposed to concurrently, leading to potential synergistic or antagonistic effects. These toxic effects on CYP enzymes in fish can have far-reaching implications for the health and survival of fish populations in pesticide-contaminated environments [94].

Atrazine has been found to affect the expression and activity of cytochrome P450 (CYP) enzymes in fish (Table 1). Studies on zebrafish have demonstrated that atrazine exposure can alter the expression of various CYP genes, such as CYP1A, CYP1B1, and CYP3A, which are involved in the metabolism of xenobiotics, including pesticides [24], [65]. Atrazine has been shown to induce CYP1A, CYP3A, and CYP7A enzymes in zebrafish liver and kidney tissues [47]. Induction of CYP enzymes can lead to increased biotransformation of xenobiotics and altered metabolism. Conversely, atrazine has also been found to inhibit the activity of certain CYP enzymes, such as CYP1A1 and CYP3A4, in zebrafish liver microsomes [47]. This inhibition can impair the metabolism of drugs and xenobiotics. Atrazine exposure decreased ATP production in zebrafish liver mitochondria, indicating impaired energy metabolism [97].

**Table 1:** Effects of atrazine on enzymes in *C. carpio* and *D. rerio*.

**Authors**	**Fish species**	**enzyme**	**Effects of atrazine**
[96], [95]	*C. carpio*	CYP450	increased
[94]	*C. carpio*	CYP450	increased
[20]	*D. rerio*	CYP450	increased
[93]	*C. carpio*	GSTs	decreased
[45]	*D. rerio*	*UDP-glucuronosyltransferases*	decreased
[22]	*C. carpio*	*UDP-glucuronosyltransferases*	decreased
[7]	*D. rerio*	SOD	increased
[84]	*C. carpio*	SOD	decreased
[7]	*D. rerio*	CAT	decreased
[84]	*C. carpio*	CAT	decreased
[87]	*D. rerio*	GPx	increased
[57]	*D. rerio*	AChE	decreased
[80]	*C. carpio*	AChE	decreased
[82]	*C. carpio*	NKA	decreased
[44]	*C. carpio*	Lysozyme	decreased
[27]	*D. rerio*	Lysozyme	decreased
[35]	*D. rerio*	Peroxidase	decreased
[90]	*D. rerio*	DNMT	decreased

Atrazine has been found to alter the expression of genes involved in energy metabolism, such as those involved in glycolysis, fatty acid oxidation, and oxidative phosphorylation [77], [78].

The exact mechanisms by which atrazine affects CYP enzymes and energy flow in zebrafish are not fully understood but may involve [77], [78], [97]:

− **Hormonal disruption:** Atrazine can bind to estrogen receptors, leading to changes in gene expression and hormonal signaling pathways that affect metabolism and energy homeostasis.
− **Oxidative stress:** Atrazine can generate reactive oxygen species (ROS), damaging cellular components and disrupting normal metabolic processes.
− **Epigenetic modifications:** Atrazine may alter epigenetic marks on gene promoters, leading to changes in gene expression and affecting energy metabolism.

In common carp, atrazine exposure has been shown to induce CYP enzyme activity, particularly CYP1A and CYP3A, which are involved in the biotransformation of organic pollutants. This increased CYP enzyme activity suggests the fish's response to detoxify and eliminate atrazine and its metabolites. However, prolonged or high-dose exposure to atrazine may overload the detoxification system, potentially causing adverse effects on the fish's health [22].

The effects of atrazine on CYP enzymes in these fish species illustrate the diverse responses of different species to pesticide exposure and emphasize the importance of understanding the specific molecular mechanisms underlying pesticide toxicity in aquatic organisms.

The mechanisms through which atrazine affects cytochrome P450 (CYP) enzymes in zebrafish and common carp involve complex interactions at the molecular level. Atrazine can act as an inducer of CYP enzymes, triggering the up-regulation of specific CYP genes in response to exposure. This up-regulation is often mediated by the activation of regulatory proteins, such as the aryl hydrocarbon receptor (AHR) pathway, which plays a central role in the transcriptional regulation of CYP genes [22], [53], [94].

In zebrafish, atrazine exposure has been shown to activate the AHR pathway (Aryl Hydrocarbon Receptor signaling pathway), leading to the up-regulation of CYP1A and other CYP genes involved in the metabolism of xenobiotics. This up-regulation reflects the fish's attempt to metabolize and eliminate atrazine and its metabolites from its system [3].

Similarly, in common carp, atrazine exposure can induce the expression of CYP1A and CYP3A through AHR-mediated pathways, enhancing the fish's capacity to metabolize and detoxify the herbicide [84]. Also, another study showed that the exposure of peripheral neutrophils of common carp to atrazine induced the mRNA expression of CYPs enzymes (CYP1A1, CYP1B1, CYP1C and CYP3A138) [84] in order to eliminate the herbicide.

Understanding these molecular mechanisms is crucial for assessing the potential impacts of atrazine and similar pesticides on fish health and for developing strategies to mitigate their adverse effects on aquatic ecosystems.

### 2.2 Glutation-S-transpherases

Pesticides, including atrazine, can also impact the activity and expression of glutathione *S*-transferases (GSTs) in fish, such as zebrafish and common carp. Glutathione *S*-transferases are a family of detoxification enzymes that play a vital role in metabolizing and eliminating a wide range of endogenous and exogenous compounds, including pesticides.

In zebrafish, exposure to atrazine has been shown to affect the expression and activity of GSTs. Atrazine can induce the up-regulation of certain GST isoforms, reflecting the fish's response to detoxify and eliminate the herbicide from its system. However, prolonged or high-dose exposure to atrazine can lead to increased oxidative stress and depletion of cellular antioxidants, potentially impacting the overall detoxification capacity of GSTs and adversely affecting zebrafish health [7].

Similarly, in common carp, atrazine exposure can influence GST activity, with the herbicide potentially acting as a substrate for GST enzymes. This may result in the conjugation of atrazine with glutathione, serving as a mechanism for detoxification and elimination. However, the sustained exposure to atrazine can disrupt the balance of cellular redox status, affecting the overall function of GSTs and potentially leading to oxidative damage in common carp. For example a decrease in GSTs was observed in *C. carpio* after exposure to atrazine [93].

### 2.3 UDP-glucuronosyltransferases (UGT)

Uridine 5′-diphospho-glucuronosyltransferases (UDP-glucuronosyltransferases, UGT) are enzymes responsible for the detoxification and elimination of various xenobiotic compounds, including pesticides, drugs, and other potentially harmful substances. They achieve this by catalyzing the transfer of glucuronic acid from the coenzyme UDP-glucuronic acid to the target compound, forming glucuronides that are more easily excreted.

Studies have shown that atrazine exposure can modulate the expression and activity of UGTs in zebrafish and common carp. For example, a study by Jones et al. [35] found that zebrafish larvae exposed to atrazine exhibited altered UGT gene expression patterns, with some UGT isoforms being up-regulated while others were down-regulated. This suggests that atrazine exposure can disrupt the normal regulation of UGTs in fish.

Other studies have also demonstrated that atrazine exposure impair UGT activity in zebrafish and common carp. For instance, Zhang et al. [98] showed that atrazine exposure reduced UGT activity and impaired detoxification capacity in zebrafish liver cells. Similarly, Fu et al. [22] found that atrazine exposure decreased UGT activity in common carp liver.

The inhibition or alteration of UGT activity and expression can have negative consequences for fish. It can disrupt the detoxification process, leading to increased susceptibility to chemical toxicity and potential accumulation of harmful substances. Additionally, impaired UGT function can affect the metabolism and elimination of endogenous compounds, such as hormones and bile acids, which play vital roles in various physiological processes [75].

### 2.4 Oxidative enzymes

Pesticides like atrazine can have toxic effects on fish, including zebrafish and common carp, by impacting oxidative stress enzymes. Oxidative stress enzymes are crucial in maintaining the balance between reactive oxygen species (ROS) production and their elimination, ultimately protecting the cells from oxidative damage [7].

Atrazine exposure has been found to induce oxidative stress in fish by disrupting the delicate balance between ROS production and antioxidant defenses. Several studies have examined the effects of atrazine on oxidative stress enzymes in zebrafish and common carp.

One example is the enzyme superoxide dismutase (SOD), which acts as the first line of defense against oxidative stress by catalyzing the conversion of superoxide radicals into less harmful hydrogen peroxide. Results of previous studies showed that long-term exposure to atrazine could increase the activity of SOD in order to protect the fish against harmful ROSs [7], [12]. On the other hand, atrazine exposure inhibited the SOD in neutrophils of the common carp [84].

Another enzyme affected by atrazine is catalase (CAT), which plays a crucial role in converting hydrogen peroxide into water and oxygen, thus preventing cellular damage [52]. Research by Blahová et al. [7] demonstrated that atrazine exposure resulted in decreased CAT activity in zebrafish, suggesting reduced ability to neutralize hydrogen peroxide. In common carp, atrazine exposure inhibited CAT in neutrophils as well [84].

Furthermore, studies have shown that atrazine exposure can affect other vital oxidative stress markers, such as glutathione peroxidase (GPx) and glutathione-*S*-transferase (GST). GPx (glutathione peroxidase) activity, an enzyme responsible for reducing harmful peroxides, was found to be increased by atrazine in zebrafish embryos, juveniles and moderately in adults [86], [87]. Meanwhile, GST, which plays a critical role in detoxifying and eliminating xenobiotics, was shown to be affected by atrazine exposure in common carp. The activity of GST was decreased significantly after exposure of the *C. carpio* to atrazine, and the expression of four GSTs isoforms transcripts (GSTK, GSTT, GSTR, and GSTM) in the liver, brain, kidney, and gill was up-regulated or down-regulated based on the tissue, concentration, and duration of the exposure [93]; Ji et al., 2020).

### 2.5 Acetylcholinesterase

Acetylcholinesterase (AChE) is an enzyme breaking down the neurotransmitter acetylcholine. Inhibition of AChE activity can disrupt normal neural function and lead to various neurotoxic effects. Atrazine exposure has been found to inhibit AChE activity in both zebrafish and common carp. A study conducted on zebrafish embryos showed that atrazine caused a significant reduction in AChE activity at different stages of development [57]. Similarly, another study on common carp reported decreased AChE activity in brain tissue after exposure to atrazine [80].

Atrazine-induced inhibition of AChE can lead to an accumulation of acetylcholine in the synaptic cleft, disrupting normal cholinergic signaling. This disruption can interfere with the transmission of nerve impulses and affect vital physiological processes regulated by the cholinergic system.

Inhibition of AChE activity by atrazine can result in various neurobehavioral effects in zebrafish and common carp. Studies have observed alterations in swimming behavior, locomotor activity, and coordination as a consequence of disrupted cholinergic signaling [57], [80]. These behavioral changes may indicate impaired neurological function.

## 3 DNA methyl transferase (DNMT)

DNA methylation, a phenomenon occurring at CG-rich segments of the DNA, is critical in normal cellular development processes due to its role in regulating gene transcription, transposon inactivation, X-chromosome inactivation, and finally, genomic imprinting. This process is mediated by an enzyme family named DNA methyltransferases (DNMTs) which catalyze the transfer of a single methyl group from S-adenosyl methionine to cytosine in DNA. Previous studies showed that atrazine exposure could decrease the DNMTs activities in zebrafish impairing several cellular processes [90]. Results of the Wang et al. [83] also showed that atrazine exposure decreased the methylation of the DNA in the liver, kidney and gills of the common carp due to down-regulation and reduction in DNMT expression.

### 3.1 Na^+^, K^+^-ATPase

Atrazine has been found to have toxic effects on the Na^+^, K^+^-ATPase enzyme (NKA) in fish. Na^+^, K^+^-ATPase is an important membrane-bound enzyme playing a critical role in maintaining cellular ion balance, cell volume and membrane resting potential. It actively pumps sodium ions (Na^+^) out of the cells and potassium ions (K^+^) into the cells, creating an electrochemical gradient across the cell membrane [36], [37].

Atrazine exposure has been shown to inhibit the activity of Na^+^, K^+^-ATPase in fish. This inhibition disrupts the normal functioning of the enzyme, leading to imbalances in intracellular ion concentrations. The inhibition of Na^+^, K^+^-ATPase can impair the regulation of ion transport and disrupt osmoregulation, which is crucial for maintaining water and electrolyte balance in fish. Atrazine-induced inhibition of Na^+^, K^+^-ATPase can disturb the balance of Na^+^ and K^+^ ions inside and outside the fish cells. This disruption can lead to changes in cell membrane potential and alter the normal physiological processes that depend on proper ion concentrations. Imbalances in ion homeostasis can negatively affect various physiological functions, such as nerve conduction, muscle contraction, and kidney function [55], [85].

Gill tissues in fish play a vital role in gas exchange and ion regulation. Atrazine exposure can directly impact the Na^+^, K^+^-ATPase activity in the gills, affecting ion transport across the gill epithelium. This can result in increased ion loss from the fish's body, leading to disturbances in osmoregulation and potential dehydration [40]. It has been previously demonstrated that atrazine exposure decreased the activity of NKA in *C. carpio* [82], [84], which could further lead to failure in osmo and ion regulation. Atrazine exposure has been associated with generating reactive oxygen species (ROS) in fish. ROS can cause oxidative damage to various cellular components, including the Na^+^, K^+^-ATPase enzyme, as an indirect effects of pesticide exposure. This oxidative stress can further impair the enzyme's activity and contribute to cellular dysfunction.

### 3.2 Immune enzymes

Atrazine, a commonly used herbicide, can have toxic effects on the immune system of fish such as zebrafish and common carp by impacting immune enzymes. The immune system in fish relies on a variety of enzymes to fight off pathogens and maintain immune homeostasis. Pesticides such as atrazine can suppress or alter the immune system of fish, making them more susceptible to diseases and infections. Atrazine exposure has been shown to impair immune responses, reduce immune enzyme activity, and lower antibody production in zebrafish and common carp [46]. Weakened immune function can compromise the fish's ability to defend against pathogens and reduce their overall fitness. Here are some examples of immune enzymes that can be affected by atrazine:

### 3.3 Lysozyme

Lysozyme is an enzyme that plays a crucial role in the innate immune system by breaking down bacterial cell walls. Studies have shown that exposure to atrazine can reduce lysozyme activity in common carp [61], [44] and zebrafish [27]. Decreased lysozyme activity makes fish more susceptible to bacterial infections.

### 3.4 Peroxidase

Peroxidase is another important immune enzyme involved in the defense against pathogens. Research has indicated that atrazine exposure reduce peroxidase activity in zebrafish [34]. This decrease in peroxidase activity weakens the fish's ability to neutralize harmful substances from invading microorganisms.

### 3.5 Immunoglobulins

Immunoglobulins, also known as antibodies, are proteins that play a critical role in the adaptive immune response. They help identify and neutralize specific pathogens. Exposure to atrazine has been found to affect the expression of immunoglobulin genes in zebrafish, potentially impairing the fish's ability to mount an effective immune response [27].

### 3.6 Complement system

The complement system is part of the innate immune response and aids in the clearance of pathogens. Atrazine exposure in zebrafish has been shown to reduce the complement system activity, suggesting a potential disruption in immune function [27].

The toxic effects of atrazine on immune enzymes can weaken the fish's immune system, making them more susceptible to infections, diseases, and other stressors. A compromised immune system can lead to higher mortality rates, reduced growth, and overall poor health.

## 4 Development and reproductive processes

Pesticides, including atrazine, can harm zebrafish and common carp. These effects can be detrimental to their health and overall well-being. Here are some key toxic effects of pesticides like atrazine on these fish species:

### 4.1 Developmental abnormalities

Exposure to pesticides during critical developmental stages can lead to various abnormalities in fish. Studies have shown that atrazine exposure can cause malformations in zebrafish and common carp embryos, affecting their growth and survival [69]. Abnormalities may include spinal deformities, craniofacial malformations, and impaired organ development [1].

Atrazine exposure can impair the growth and survival of fish embryos and larvae. Research has demonstrated that atrazine can cause reduced hatching success, delayed hatching, and decreased survival rates in zebrafish and common carp [1], [12]. It can also negatively affect growth parameters, including body length and weight [41].

Atrazine exposure during early development can have neurotoxic effects on zebrafish and common carp larvae. Studies have shown that atrazine can disrupt the normal development and function of the nervous system, leading to impaired behavior, altered locomotor activity, and changes in neurotransmitter levels [81], [42], [18], [1].

As mentioned earlier, atrazine is an endocrine disruptor and can interfere with hormonal regulation. During the critical stages of embryonic and larval development, exposure to atrazine can disrupt the normal balance of hormones, affecting processes such as differentiation, growth, and maturation [64]. This disruption can have long-lasting effects on the health and development of zebrafish and common carp.

### 4.2 Reproductive toxicity

Pesticides such as atrazine can disrupt the reproductive capabilities of fish. They may interfere with the production of hormones involved in reproduction, leading to reduced fertility, altered sex ratios, and impaired gamete quality. In zebrafish, atrazine exposure has been linked to changes in gonadal development and decreased fertility [13].

Atrazine is known to be an endocrine disruptor, meaning it interferes with the normal functioning of the endocrine system, including reproductive hormone regulation. It can mimic or block the actions of natural hormones, leading to imbalances in the reproductive system.

Atrazine has also exhibited estrogenic activity as well, meaning it can mimic the effects of the estrogen hormone. In zebrafish and common carp, atrazine exposure can increase estrogen production, which can lead to disrupting the normal hormone balance [88], [94]. This unbalancing can adversely affect reproduction, reproductive development, and overall reproductive health.

Atrazine exposure can also interfere with the production and regulation of testosterone, an essential male sex hormone. Studies have shown that atrazine can decrease testosterone levels in zebrafish and common carp [88], [94]. Reduced testosterone can affect reproductive behavior, fertility, and sperm quality in males.

Pesticide exposure, including atrazine, has been shown to disrupt gonadal development in fish species. In zebrafish and common carp, atrazine exposure can result in abnormal gonad development, including changes in size, shape, and structure [13]. These alterations can negatively impact reproductive function and fertility.

Embryonic exposure of zebrafish to atrazine decreased spawning and caused morphological alterations in offspring (Wirbsky et al., 2016b).

Atrazine exposure can affect oocyte development, particularly in female fish. Oocytes are the developing eggs within the ovaries. Studies have demonstrated that atrazine exposure can lead to abnormal oocyte growth, impaired maturation, and decreased egg production in zebrafish and common carp [6], [88]. These effects can disrupt the reproductive cycle and reduce reproductive success.

## 5 Genotoxicity

Pesticides can induce genotoxic effects, causing damage to the DNA of fish. Atrazine exposure has been associated with genetic mutations and chromosomal aberrations in zebrafish and common carp [78], [77]. Genotoxicity can lead to long-term health issues and affect the population's genetic diversity and stability.

Genotoxicity refers to the ability of a substance, such as atrazine, to damage genetic material (DNA) within cells. In the case of zebrafish and common carp, exposure to pesticides like atrazine can lead to genotoxic effects as follows:

### 5.1 DNA damage

Atrazine can directly damage the DNA of zebrafish and common carp, resulting in various types of DNA alterations, including single-strand breaks (a break in one of the two DNA strands), double-strand breaks (breaks in both DNA strands), and DNA adducts (chemical modifications to the DNA structure). These types of damage can disrupt the integrity and functioning of the genetic material within cells [99], [79].

### 5.2 Micronucleus formation

Micronuclei are small, extra nuclei that can form during cell division due to DNA damage or chromosome breakage. Studies have shown that exposure to atrazine can increase the micronuclei formation in zebrafish and common carp. These micronuclei can contain damaged chromosomal fragments or whole chromosomes, indicating genotoxic effects [58].

### 5.3 DNA repair mechanisms

Zebrafish and common carp possess DNA repair mechanisms that help to fix damaged DNA. However, exposure to atrazine can overwhelm these repair processes, leading to incomplete or inaccurate repair, further contributing to an accumulation of genetic damage within the cells [9], [56].

### 5.4 Gene expression

Atrazine exposure can lead to changes in the expression of specific genes in zebrafish and common carp. Gene expression refers to the process by which information stored in DNA is used to produce functional proteins. Pesticides can interfere with this process by either up-regulating (increasing) or down-regulating (decreasing) the expression of specific genes [30], [31], [70].

Atrazine can disrupt the expression of genes involved in hormone signaling pathways. For instance, in zebrafish, exposure to atrazine has been found to up-regulate the expression of aromatase, an enzyme responsible for converting androgens to estrogens. This up-regulation can lead to an imbalance in the estrogenic signaling pathway. In common carp, atrazine exposure has been shown to influence the expression of androgen and estrogen receptors, which can affect reproductive processes [88].

Atrazine exposure during critical developmental stages can affect the expression of genes involved in embryonic development. In zebrafish, exposure to atrazine has been linked to altered expression of genes related to neural development, such as Pax6 and Shh. These genetic alterations can result in abnormalities in brain and spinal cord development. Similarly, atrazine exposure in common carp has been associated with altered expression of genes involved in skeletal development, such as collagen genes, leading to skeletal malformations [2].

Pesticides like atrazine trigger the activation of detoxification mechanisms. Zebrafish and common carp respond by up-regulating genes involved in detoxification pathways, such as cytochrome P450 enzymes. These enzymes help break down and eliminate the pesticide from the body. Atrazine exposure has been shown to influence the expression of these detoxification genes, potentially affecting the organism's ability to metabolize and eliminate the pesticide effectively.

Atrazine exposure can induce oxidative stress, leading to the activation of genes involved in antioxidant defense mechanisms. For example, zebrafish and common carp may up-regulate genes encoding antioxidant enzymes like superoxide dismutase and catalase. These enzymes help counteract the reactive oxygen species (ROS) generated during oxidative stress [24].

Furthermore, it is been revealed that exposure to atrazine increased the expression levels of the core components of necroptosis (RIPK1, RIPK3 and MLKL) and inflammatory factors (TNF-α, NF-κB, iNOS, COX-2, IL-1β and PTGE) in common carp [96], [95].

Atrazine exposure can affect the expression of these genes, impacting the organism's ability to mitigate oxidative damage.

Also, it has been revealed that atrazine exposure activates Caspase3 and induces apoptosis in neutrophils of the common carp by changing the expression of mitochondrial pathway factors (Bcl-2, BAX and Caspase9) and death receptor pathway major genes (TNF-α TNFR, Fas, FasL, and Caspase8) [84]. The latter could impair the fish’s immune function of the fish and endanger it against pathogens and diseases.

### 5.5 miRNAs

MiRNAs are small, short RNAs that can regulate post-transcriptional gene expression by controlling the mRNAs. This epigenetic process could play a role in multiple cellular functions, including xenobiotic responses [5]. It has been revealed that embryonic exposure of zebrafish to atrazine altered miRNAs associated with angiogenesis, cancer and neurodevelopment [89].

## 6 Disruption of neurological function

Pesticides can also impact the nervous system of fish. Atrazine exposure has been linked to neurotoxic effects, including altered behavior, impaired locomotion, and changes in neurotransmitter levels in zebrafish [10]. Neurological disruption can affect normal functioning, foraging abilities, and predator avoidance in fish. Atrazine is known to interfere with the production of neurotransmitters, responsible for transmitting signals in the brain. This disruption can lead to impaired neurological functions in fish, affecting their behavior, movement, and sensory perception. When it comes to the neurotoxic effects of atrazine on zebrafish and common carp, several specific effects that have been observed:

### 6.1 Behavioral changes

Exposure to atrazine can lead to alterations in fish behavior. Zebrafish and common carp may display changes in locomotor activity, such as decreased swimming speed, increased time spent in immobile states, or disrupted schooling behavior [2].

### 6.2 Impaired sensory perception

Atrazine can affect the sensory system of fish, including their ability to detect and respond to stimuli, resulting in reduced sensory perception, affecting their ability to locate food, respond to predators, or navigate through their environment [81]. It has been demonstrated that the motor integration ability of the zebrafish could damage after exposure to atrazine which can disturb the development of sensory neurons and the innervations of muscles resulting in the failure of many body activities [51], [81].

### 6.3 Altered neurotransmitter levels

Atrazine exposure can disrupt the balance of neurotransmitters in the brain of zebrafish and common carp. Neurotransmitters are chemical messengers that allow communication between nerve cells. Changes in neurotransmitter levels can lead to disturbances in neuronal signaling, potentially impacting various physiological processes. For example, after exposure of zebrafish to atrazine, acetylcholine (neuron-muscle synaptic transmitter) activity was disturbed due to change in the activity of AChE resulting in impair muscle activity [81].

### 6.4 Morphological changes in the brain

Studies have shown that atrazine exposure can cause structural changes in the brains of fish, including alterations in the size and organization of specific brain regions involved in various functions, such as sensory processing, learning, and memory. A study showed significant alterations in craniofacial cartilage development in embryos of *D. rerio* [76].

## 7 Histopathology

Atrazine is a widely used herbicide that can potentially have adverse effects on aquatic organisms [26]. In zebrafish and common carp, exposure to atrazine can lead to various histopathological changes in their organs and tissues (Table 2).

**Table 2:** Histopathological effects of atrazine in zebrafish and common carp.

**Tissue/Organ**	**alterations**	**Authors**
Liver	hepatocyte degeneration, inflammation, necrosis, and fibrosis	[33], [59], [92]
Kidney	tubular degeneration, interstitial inflammation, and nephron damage	[59], [91]
Gill	epithelial hyperplasia, lamellar fusion, and lamellar edema	[59], [66]
Reproductive organs	reduced spermatogenesis, interstitial cell hypertrophy, and testicular degeneration	[88]

### 7.1 Liver

Atrazine exposure has been shown to cause liver damage in both zebrafish and common carp. Histopathological changes may include hepatocyte degeneration, inflammation, necrosis, and fibrosis [33], [92], [59].

### 7.2 Kidneys

Atrazine can also affect the kidneys of zebrafish and common carp. Histopathological effects may include tubular degeneration, interstitial inflammation, and nephron damage [91], [59].

### 7.3 Gills

As aquatic organisms, zebrafish and common carp are particularly vulnerable to pesticides in their gills. Atrazine exposure can cause gill tissue damage, including epithelial hyperplasia, lamellar fusion, and lamellar edema [66], [59].

### 7.4 Reproductive organs

Atrazine has been associated with reproductive toxicity in fish. In zebrafish, it may lead to histopathological changes in the testes, such as reduced spermatogenesis, interstitial cell hypertrophy, and testicular degeneration. In common carp, atrazine exposure may affect ovarian follicles, leading to degeneration and disruption of normal reproductive processes [88].

## 8 Bioaccumulation of atrazine

Zebrafish is not used in food and fishery industries, and its value is more from ecological, laboratory and biological aspects. Therefore, in this section only the bioaccumulation of atrazine and its effects in common carp are investigated.

Atrazine is a lipophilic and hydrophobic compound that it tends to accumulate in fatty tissues and biomembranes. In common carp, atrazine has been shown to bioaccumulate in various tissues, including fat tissue (especially in adipose tissue and liver), muscle tissue (including edible portions of the fish), and gill tissue (probably because of direct contact with the surrounding water) [74], [96], [95].

The bioaccumulation of atrazine in common carp can occur through various routes, including [74]:

− Environmental exposure: Common carp can absorb atrazine from contaminated water, sediments, or food sources.
− Food chain accumulation: Atrazine can accumulate in aquatic organisms through the food chain, with larger fish like common carp consuming smaller fish or other organisms that have previously accumulated atrazine.

The bioaccumulation of atrazine in common carp poses potential health risks for human consumers, particularly those who consume the fish regularly or in large quantities. Possible hazards include [60], [72], [74]:

− **Cancer risk:** Long-term consumption of atrazine-contaminated fish may increase the risk of cancer due to the compound's endocrine-disrupting properties and potential mutagenicity.
− **Reproductive and developmental effects:** Atrazine has been linked to reproductive and developmental toxicity in animal studies. Consuming atrazine-contaminated fish may pose a risk to human reproduction and fetal development.
− **Neurological effects:** Exposure to atrazine has been associated with neurological problems, including changes in behavior, cognition, and neurodevelopment.
− **Endocrine disruption:** Atrazine can bind to estrogen receptors, potentially leading to hormonal imbalances and disruptions in human physiology.

## 9 Discussion

The increasing use of pesticides has become one of the problems of protecting the environment. Some of these pesticides are persistent and enter aquatic environments through rain, surface runoff, or urban and agricultural effluents. These compounds damage different organisms in aquatic ecosystems [16].

Atrazine herbicide is one of the most common herbicides, used in agriculture and urban activities to kill broadleaf weeds. Studies have shown that atrazine is found in many surface and ground waters. This compound is known as an endocrine disruptor compound, which causes many disorders in the endocrine system of aquatic animals [19]. In addition to disrupting the endocrine system, various studies on the effects of atrazine on cells and tissues, DNA, gene expression, and others, in different fish, including zebrafish and common carp, show that this herbicide can cause disruption or damage to different parts of the body of these fish [84]. Among these changes, tissue damage to the gills, liver and kidney can be mentioned. Also, alterations in the activity level of different enzymes and changes in gene expression of different proteins, including important cellular enzymes, are other effects of atrazine in these two species of fish [98], [30].

Several studies indicated that exposure to atrazine resulted in notable alterations in both the morphology and behavior of zebrafish and common carp. Atrazine exposure in zebrafish led to an elevated occurrence of abnormal swimming behaviors, decreased levels of activity, and diminished appetite. These observed changes align with previous research in different fish species, demonstrating that atrazine exposure can interfere with motor function and behavior [29], [54]. Similarly, common carp exposed to atrazine exhibited significant behavioral alterations such as reduced swimming speed and increased unpredictable swimming patterns. These findings imply that atrazine could have widespread effects on the behavior of aquatic organisms, potentially disrupting their social interactions and habitat use.

Previous research revealed that atrazine exposure led to both behavioral and morphological changes in both species. These findings align with previous research indicating that atrazine exposure can result in developmental toxicity in fish [1]. The developmental toxicity linked to atrazine is believed to be caused by the disruption of hormone regulation [43].

The variation in sensitivity to atrazine exposure between zebrafish and common carp is likely attributed to differences in their metabolism and physiology. Zebrafish, a genetically tractable model organism, has been extensively researched for their capacity to absorb and process xenobiotics [17]. In contrast, common carp, a more ecologically relevant species, often encounters pesticides in agricultural runoff. The differences in sensitivity between these two species emphasize the significance of studying multiple species when evaluating the environmental consequences of pesticides. Also, it is important to note that the specific impacts and severity of pesticide effects on fish can vary depending on factors such as the type of commercial formulation, its concentration and duration of exposure.

A comprehensive understanding of the effects of pollutants, including the atrazine herbicide, is essential, which leads to a better understanding of the effects of these compounds on aquatic organisms. This can be the background for understanding the effects of these substances on humans (due to the similarity of many cases between humans and zebrafish). On the other hand, a complete understanding of the various effects of atrazine (and other pollutants) can help formulate the principles and rules limiting the use of these compounds. Implementing environmentally friendly agricultural practices and adopting integrated pest management strategies can help reduce the negative impacts of atrazine on non-target aquatic organisms.

## Declaration of Competing Interest

The authors declare that they have no known competing financial interests or personal relationships that could have appeared to influence the work reported in this paper.

## Data Availability

No data was used for the research described in the article.
