# Supplementary material for: A review on toxic effects of pesticides in Zebrafish, Danio rerio and common carp, Cyprinus carpio, emphasising Atrazine herbicide
Source: Toxicol Rep. 2024 Jul 14;13:101694. doi: 10.1016/j.toxrep.2024.101694 (PMC11314875; doi:10.1016/j.toxrep.2024.101694)
Supplement: Supplementary file 1 — Supplementary material [file mmc1.docx]

**Supplementary material**

Trade names for atrazine include A 361; Aatrex; Akticon; Aktikon; Aktinit; Argezin; Atranex; Atrataf; Atrazin; Atrazine; ATZ; Azoprim; Cekuzina-T; CET; Chromozin; Cyazin; Fogard; G 30027; Gesaprim; Griffex; Hungazin; Maizina; Mebazine; Oleogesaprim; Oleogesaprim 200; Primatol; Radazin; Triazine A 1294; Vectal; Wonuk; Zeapos; Zeazin; Zeazine etc. (NCBI, 2024).
